# An improved YOLOv5s-based method for detecting rice leaves in the field

**DOI:** 10.3389/fpls.2025.1561018

**Published:** 2025-05-08

**Authors:** Cheng Zhou, Caohang Zhou, Lili Yao, Yagang Du, Xin Fang, Zhangbin Chen, Chengliang Yin

**Affiliations:** ^1^ School of Information Engineering, Huzhou University, Huzhou, China; ^2^ Institute of Agricultural Engineering, Heilongjiang Academy of Land Reclamation Sciences, Haerbin, China

**Keywords:** rice, leaf tip detection, YOLOv5S, small targets, attention mechanism

## Abstract

**Introduction:**

The number of rice leaves largely reflects the growth stage and health status of rice. However, the current rice leaf counting method is time-consuming and laborious, with low accuracy and poor efficiency, which is difficult to meet the needs of rice growth monitoring.

**Methods:**

This study proposes a field rice leaf detection method based on an improved YOLOv5s model. First, we added a high-resolution layer and removed the original low-resolution detection layer, using the K-Means++ clustering algorithm to reset the anchor box sizes, enhancing the model’s ability to identify small leaf tip targets while reducing the number of parameters. Second, we introduced a coordinate attention mechanism (CA) to strengthen focus on leaf tip features under weed interference and leaf occlusion conditions. Finally, we employed a content-aware reassembly of feature (CARAFE) upsampling operator to enhance the detail reconstruction capability of leaf tip features.

**Results and discussion:**

Experimental results showed that the improved rice leaf tip detection model achieved precision, recall, and mean average precision rates of 93.7%, 87%, and 93.5%, respectively, with a parameter count of 5.02 million (M), improving by 6.5%, 22.1%, and 18.5% compared to the YOLOv5s baseline model, while reducing the parameter count by 28.4%. The improved model effectively reduced the missed detection rate of rice leaves and enhanced the accuracy and robustness of field rice leaf tip detection, providing strong technical support for rice phenotype feature extraction and growth monitoring.

## 1 Introduction

Rice, as one of China’s major staple crops, plays a crucial role in enhancing agricultural sustainability and improving both yield and quality (Chen et al., 2021b). Plant phenotypic analysis has become an important tool for monitoring crop growth dynamics in modern agricultural research, which can accurately evaluate the physiological and developmental state of crops (Xiong et al., 2021). Leaf number is a representative agronomic trait of rice phenotype, which directly determines the photosynthetic efficiency of rice and reflects the growth and development stage of rice. It is an important indicator of crop health, nutritional status and final yield (Chen et al., 2021a; Bir et al., 2024). Leaf counts not only reflect the growth status of individual rice, but also provide an important reference in yield prediction, pest and disease monitoring, and fertilizer management of crop populations. Traditional leaf counting mainly relies on manual labor, which is not only time-consuming and inefficient in large-scale agriculture, but also affected by the subjective judgment of the counters. Although it can provide some data support, there are obvious limitations, and it is difficult to meet the demand for high-frequency and large-sample data in modern agriculture. Therefore, how to use advanced technology to realize the automation and precision detection and counting of the number of rice leaves has become a key topic in the research of smart agriculture.

In recent years, with the development of deep learning, Convolutional Neural Network (CNN) (Cong and Zhou, 2022) has been widely used in agricultural fields such as plant phenotypic feature extraction (Murphy et al., 2024), yield prediction (Van Klompenburg et al., 2020), and pest detection (Liu and Wang, 2021). CNN-based target detection algorithms have also shown good results in leaf counting and detection tasks in crops such as rice, wheat, corn and sorghum (Farjon et al., 2021). Miao et al. (2021) proposed a two deep learning-based method for automatic counting of maize and sorghum leaves: regression-based whole-plot analysis and Faster R-CNN (Faster Region-Based Convolutional Neural Net-work)-based leaf detection, in which the Faster R-CNN worked both in the occluded and visible viewpoints best reached 78% and 88%. Li et al. (2023) presented a self-supervised plant phenotyping method combining domain adaptation and 3D plant model simulation for leaf counting in wheat at seedling stage. The Faster-RCNN model combined with CycleGAN technique performs best on a diverse test dataset from five countries and achieves 94% leaf counting accuracy. Compared to the traditional two-stage target detection algorithm Fater R-CNN (Ren et al., 2017) based on region suggestion and classification regression, The single-stage detection algorithm YOLO (Shen et al., 2024) has significant advantages in terms of speed. It is not only suitable for real-time detection tasks, but also capable of maintaining high detection accuracy in complex backgrounds (Jiang et al., 2022). Hou et al. (2024) proposed a fast detection method for leaf count in wheat seedling stage based on improved YOLOv8, which improves the detection accuracy in complex field environments by fusing the coordinate attention mechanism and the small target detection layer, and the accuracy of leaf count and the average detection accuracy reach 91.6% and 85.1%, and can adapt itself to different lighting conditions to realize the efficient leaf detection. Xu et al. (2023) proposed a leaf counting method for UAV RGB images of corn seedlings combining semi-supervised learning and deep learning, using SOLOv2 and YOLOv5x networks for two-stage detection. In this method, YOLOv5x achieves 69.4% and 72.9% accuracy for counting fully expanded leaves and newborn leaves. Ning et al. (2024) proposed a lightweight corn leaf detection and counting method based on improved YOLOv8, which enhances the feature extraction and information fusion capabilities of the model by introducing StarNet network to replace the YOLOv8 backbone network and combining convolutional and attentional fusion module (CAFM) with an average detection accuracy of 97.5%. Vishal et al. (2020) used YOLOv3 to detect the leaf tips of potted rice and realized the automatic counting of rice leaves, and the final detection accuracy reached 82%. Chen et al. (2024) used YOLOv5m model combined with CBAM-CPN to extract phenotypic parameters for single tiller rice, in which YOLOv5m was used for single tiller rice leaf detection with an average detection accuracy of 91.17%.

Most scholars focus on potted crops in laboratories or seedlings with relatively less occlusion. Research on field rice leaf detection remains limited, and detecting leaf tips in field rice presents several challenges: 1) In real field environments, detection performance is greatly affected by natural factors such as lighting, weather, and weed backgrounds. 2) As the growth process advances, the morphological and color characteristics of rice leaves change significantly, with more severe occlusion between leaves, which exponentially increases the difficulty of detection. 3) Existing object detection algorithms are mainly designed for the COCO general dataset, which is insufficient to meet the needs of rice leaf detection. Therefore, this study focuses on field rice from the transplantation stage to the pre-heading stage. Based on the YOLOv5s model, improvements and optimizations are made in three areas: detection layers, attention mechanisms, and upsampling modules. These enhancements aim to improve the accuracy and robustness of field rice leaf tip detection, providing more efficient algorithmic support for rice growth monitoring systems and promoting the development of agricultural intelligence.

## 2 Materials and methods

### 2.1 Data collection

**Figure 1:**
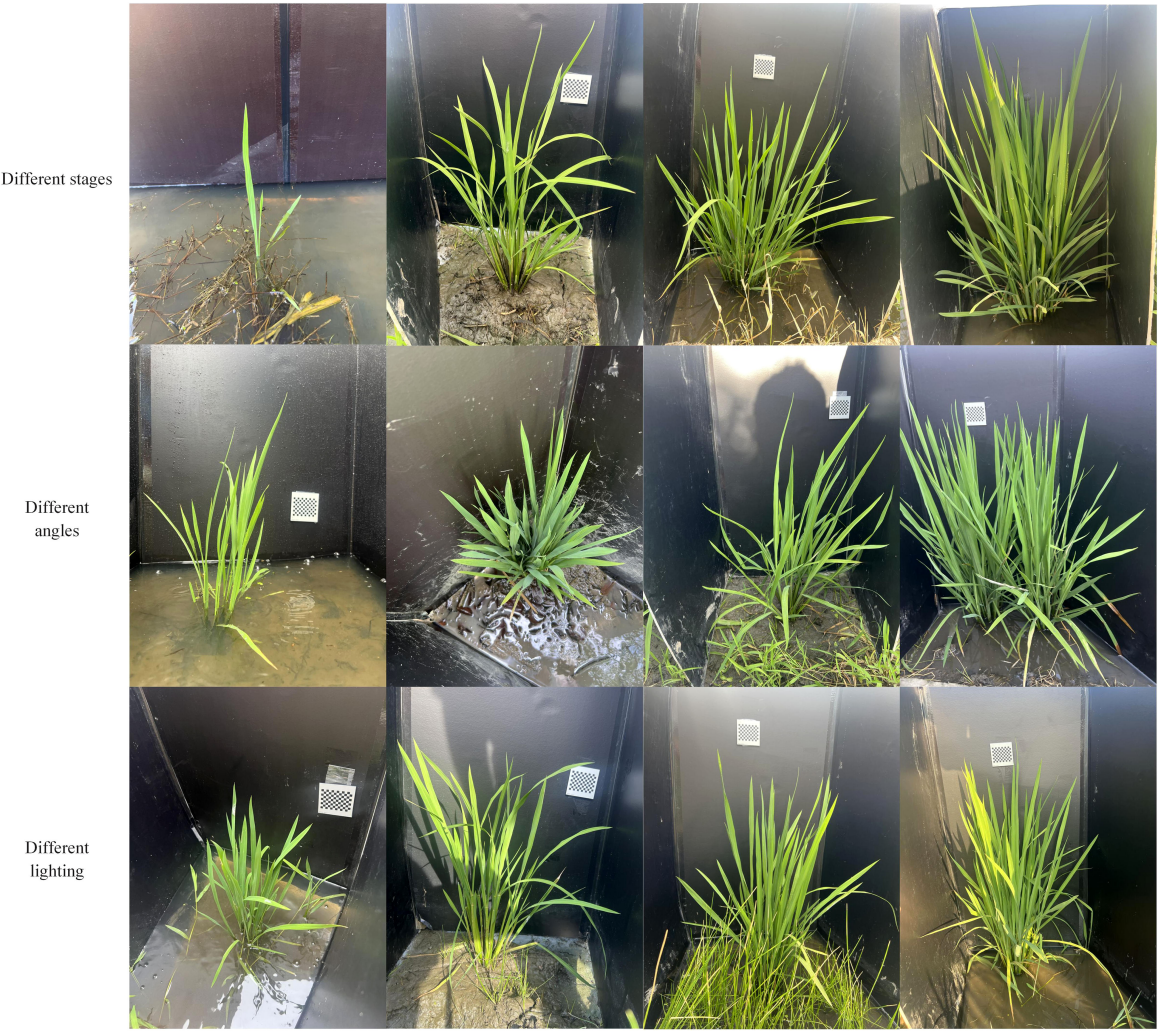
Example images from the rice data set.

In this study, rice plants were selected as the research subjects, with data collected from two experimental fields located in Huzhou, Zhejiang Province: one in Balidian Town, Wuxing District, and the other in Shanlian Town, Nanxun District. The data collection was conducted using a smartphone camera. The dataset includes three rice varieties: Chunyou927, Jiayouzhongke6, and Jianong4, covering various leaf stages from transplantation to heading. To better meet the requirements of subsequent rice growth monitoring, different smartphones were deliberately selected to capture images from multiple angles, with the background of the rice plants intentionally blocked. Additionally, different weather and lighting conditions were considered during the collection process. In total, 1,200 images of rice plants were collected. **Figure 1** shows examples of images from the rice dataset. As seen in **Figure 1**, in addition to the listed conditions, there are also complex backgrounds with weeds.

### 2.2 Dataset creation

Compared to the leaves of other broadleaf plants, rice leaves are typically sword-shaped, with long blades and distinct leaf tips (Jia et al., 2024). The leaf tip is the foremost and sharpest part of the rice leaf, characterized by clear geometric features (Zhu et al., 2021). The overlapping and crossing phenomenon between rice leaves is common in the field, making it complex and challenging to use entire leaves as features for visual identification. Since the leaf tip is located at the front end of the leaf, it is easier to identify individually, even when leaves are dense or overlapping. Therefore, this study uses the leaf tip as the feature for leaf counting. The leaf tips were manually annotated as point labels using the Labelme tool, and a 60×60pixel rectangular annotation box was generated around each point label. This approach significantly reduces the workload compared to directly drawing rectangular boxes. Additionally, using fixed-size rectangular boxes centered on point labels ensures that the model focuses on the characteristics of the leaf tip, reducing background interference, which helps the model more accurately learn leaf tip features, improving the effectiveness of leaf tip detection.

**Figure 2:**
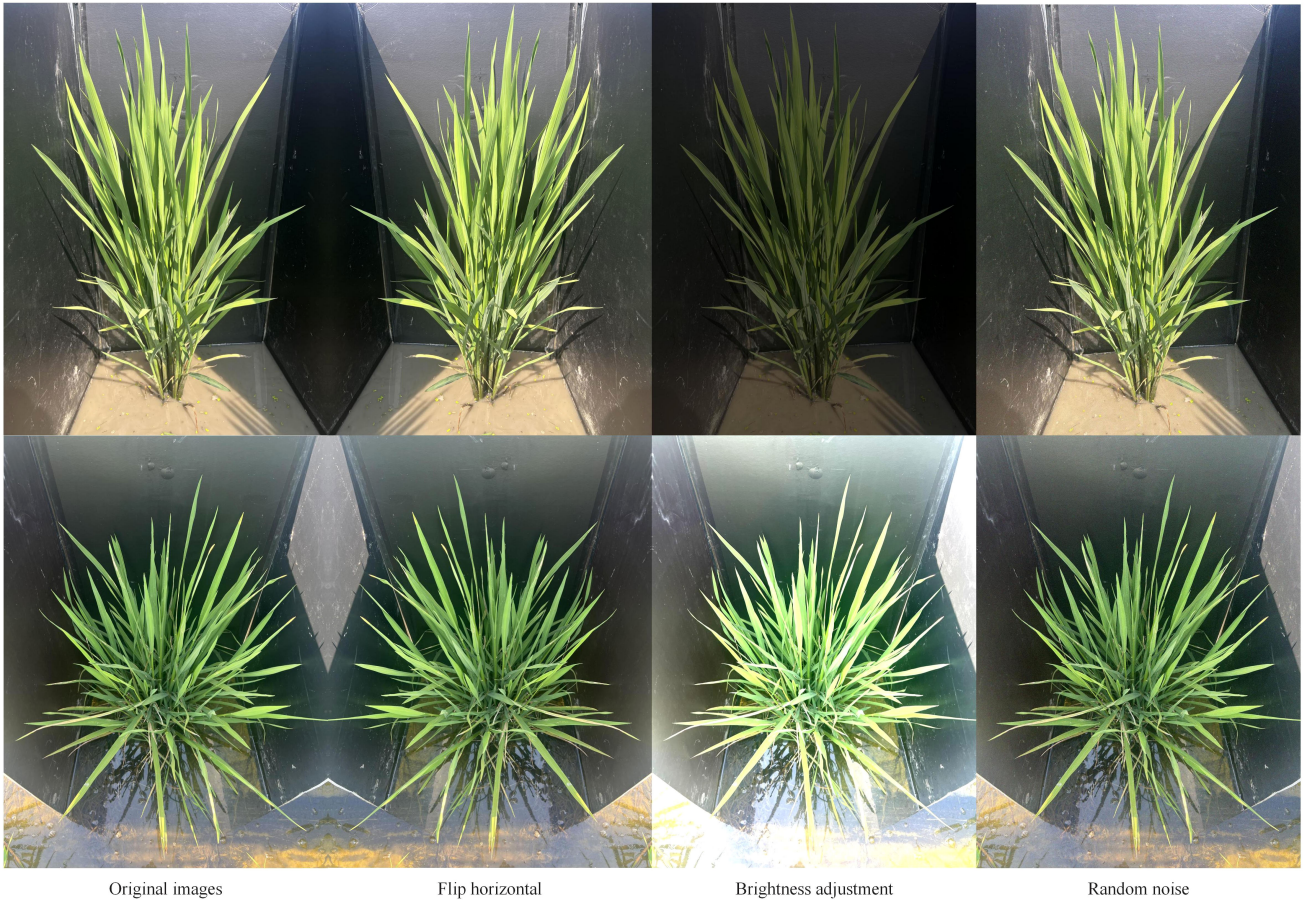
Example images of different expansion methods.

In order to further expand the dataset and increase the diversity and generalizability of the model, the study expanded the data from 1200 original images, with horizontal inversion, luminance correction, and random noise being the main methods used. Examples of images with different expansion methods are shown in **Figure 2**. The total number of final dataset after expansion is 3600 pictures, and the number of leaf tip labels is 141411. The dataset is split into training, validation and test sets in an 8:1:1 ratio finally.

### 2.3 Improving the YOLOv5s detection model

#### 2.3.1 Design of a single high-resolution detection layer

When performing the task of detecting and counting rice leaf tips, because of their extremely small size and slender morphological features, the actual length and width are usually only less than 8 mm, and the high-resolution images are scaled under the influence of the model input image size (Yu et al., 2024), making the final leaf tip size only 5×5 to 10×10 pixels, and this extremely small size makes the leaf tips easily masked by the complex background. Therefore, how to effectively detect these extremely small leaf tip targets becomes the key issue in this study.

In order to solve the challenge of small targets in rice leaf tip detection, this study proposes a single high-resolution detection layer design, which firstly introduces a high-resolution detection layer P2 into the YOLOv5s model (Zhang et al., 2022), while removing the original low-resolution detection layers P3, P4, and P5, in which the resolutions of the feature maps corresponding to P2, P3, P4, and P5 are respectively 160×160, 80×80, 40×40, and 20×20. The rice leaf tip labeling frames in the training data were then analyzed using the K-means++ clustering algorithm (Baldassi, 2022) and the preset anchor frame sizes that were more in line with the distribution of the leaf tip mini-objects were redesigned. The specific improvements are as follows:

First, a new feature fusion enhancement module is added to the original YOLOv5 network structure, which generates a P2 detection layer based on a 160×160 high-resolution feature map through 1×1 convolution, up-sampling and feature splicing operations. The high resolution feature map can retain deeper and highly semantic information, this high resolution feature map allows the model to detect at a smaller scale and can provide more pixels and richer detail information in the region of small targets, which enables the model to more effectively detect the boundaries, contours, and fine details of small objects.

Then by removing the low-resolution P3, P4, and P5 detection layers, the model avoids the problem of detail loss for small targets on low-resolution feature maps. Since small targets, such as rice leaf tips, account for a small percentage of the image, fine features may be blurred or covered by other noise information. Therefore removing these layers of low-resolution layers makes the model more focused on the details of high-resolution feature maps, reducing the loss of information on small targets. At the same time, the simplified structure not only makes the computational resources of the model more focused on the detection of small targets, better learning and adapting to the feature patterns of leaf tip small targets, but also reduces the model parameter count.

In addition, the traditional YOLOv5 model uses the K-Means algorithm to cluster the label box sizes from the COCO dataset in order to determine the preset anchor boxes, however, these preset frames are not applicable to the rice leaf tip dataset in this study. In this study, the K-Means++ algorithm is applied to cluster all the leaf tip bounding boxes to obtain three preset anchor frames. The K-Means++ algorithm improves the selection of initial clustering centers, effectively avoiding the local optimization issues caused by the randomness in the traditional K-Means algorithm (Ahmed et al., 2020), and can better adapt to the actual dimensions and shapes of the leaf tips of the rice to boost the model detection accuracy and convergence speed. The three preset anchor box sizes are [7,9], [13,10] and [17,11].

#### 2.3.2 Incorporating CA attention mechanism

**Figure 3:**
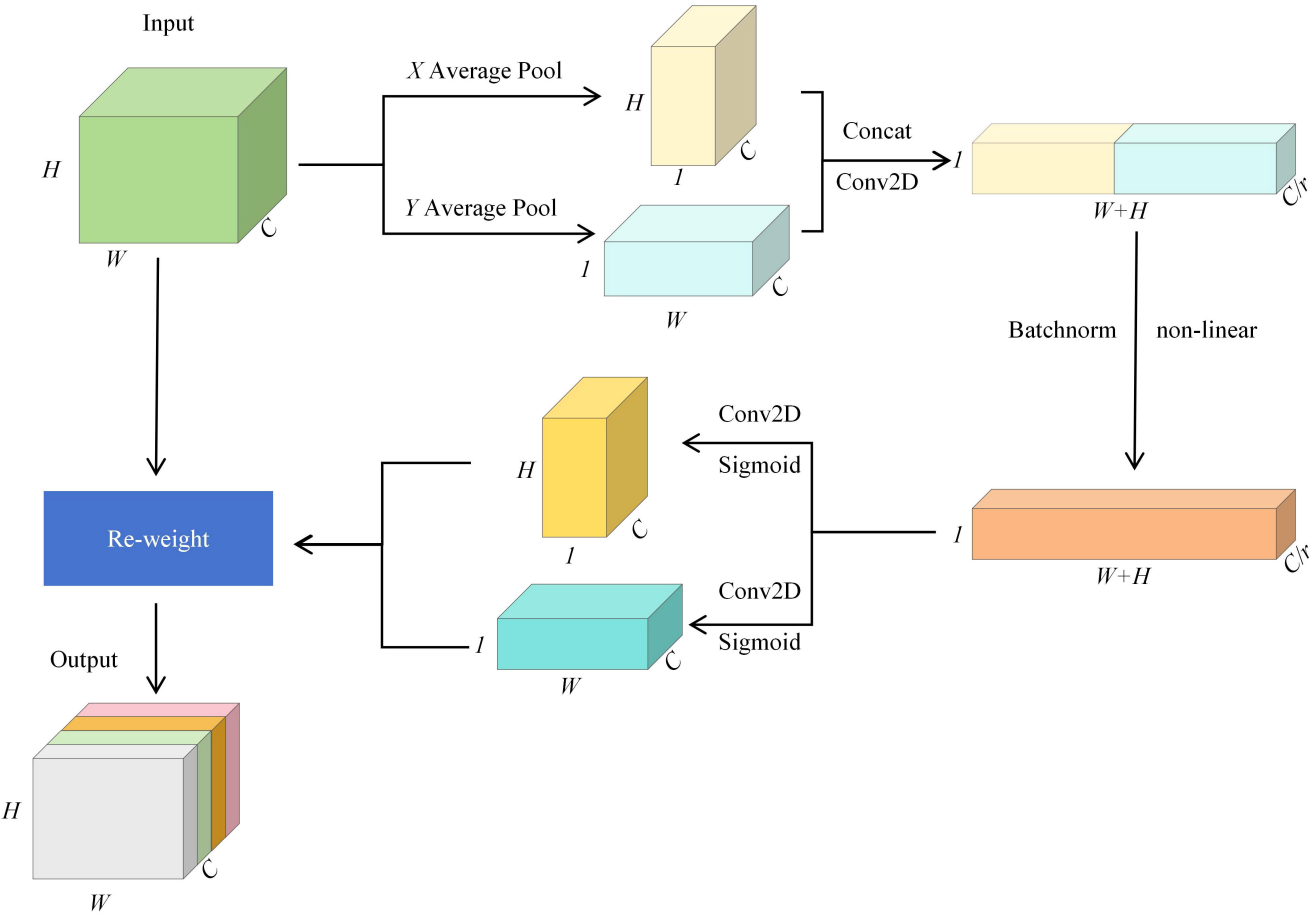
CA block structure.

In the rice leaf tip target detection task, rice leaves grow more densely and shade each other, and the leaf tip features are easily masked by other leaves, and the model may face the problems of detail loss and inaccurate target localization. To tackle this problem, this study adds a coordinate attention mechanism CA (Hou et al., 2021) between the feature fusion enhancement module and the prediction head to enhance the feature representation and spatial localization ability of the model for very small targets. **Figure 3** shows the structure of the CA attention mechanism module, which is accomplished through two steps: spatial information encoding and coordinate attention generation.

In the spatial information coding stage, it is assumed that The input feature map has dimensions of C×H×W, where C stands for the number of channels, H denotes the height, and W represents the width. To extract spatial information in the height direction Y and width direction X, the feature map is initially processed using global average pooling. Along the X and Y directions, the feature maps are encoded in the width and height dimensions to generate feature maps F_h_ and F_w_ of size C×H×1 and C×1×W, respectively.

In the coordinate attention generation stage, the two generated feature maps are first concatenated along the channel dimension, then a 1×1 convolution is applied to reduce the channel size. Batch normalization and a nonlinear activation function are then applied, forming an intermediate feature map F with a size of C/r×(W+H)×1, where r represents the channel compression ratio. The feature map is subsequently divided into two maps along the height and width dimensions. Another 1×1 convolution is applied to recover the original channel dimension C, and the Sigmoid activation function (Narayan, 1997) is applied to compute the coordinate attention weights *A_w_* and *A_h_* for the two directions. Finally they are multiplicatively weighted on the input feature map X, resulting in an output feature map of size C×H×W but weighted by coordinate attention.

#### 2.3.3 Incorporating the CARAFE upsampling operator

The traditional nearest-neighbor interpolation up-sampling method of YOLOv5 relies on a fixed interpolation rule and a limited sensory field, which is difficult to be flexibly adjusted according to the actual features of the image; this method can only utilize the nearest pixels for interpolation, which makes it difficult to capture the contextual information of a larger region, resulting in the loss of spatial details and semantic information, which in turn affects the detection accuracy (Shen et al., 2023). To address this challenge, this study introduces the CARAFE upsampling module (Wang et al., 2019) to substitute the original upsampling module based on nearest neighbor interpolation.

**Figure 4:**
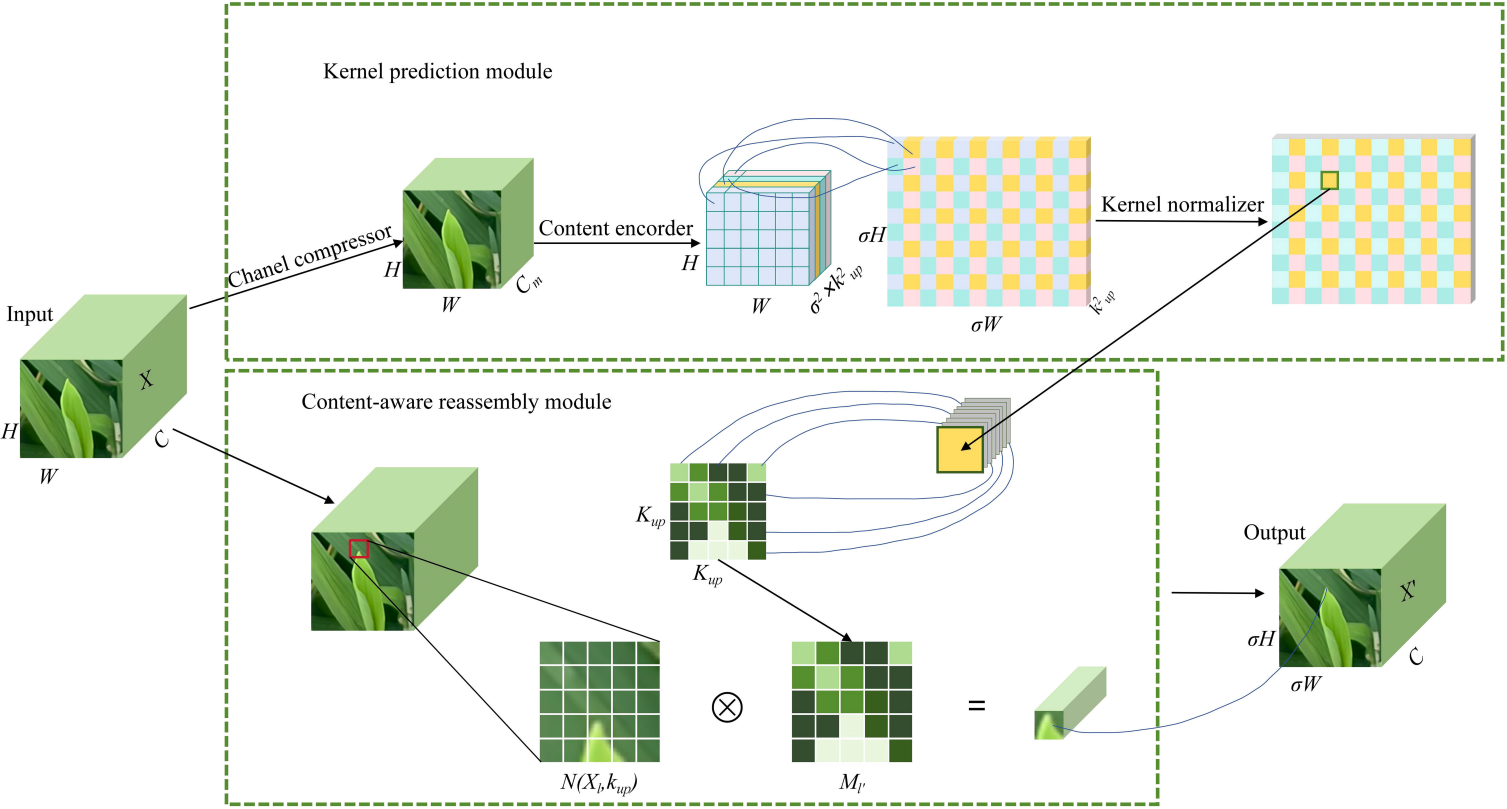
CARAFE block structure.

In CARAFE module, feature up-sampling is regarded as the process of feature reorganization, which primarily consists of the up-sampling kernel prediction module and the feature reorganization module, with the structure illustrated in **Figure 4**.

In the up-sampling kernel prediction module, the input feature map X is assumed to have a size of C×H×W, where C denotes the channel count, while H and W represent the height and width of the feature map. First, the input feature map X undergoes channel compression, reducing the number of channels from C to a smaller dimension C_m_. Then, the corresponding reorganization kernel M_l′_ is generated according to the local region features of each position in the compressed feature map. The kernel size is σ^2^×k^2up^, where σ is the upsampling ratio, and K_up_ is the size of the reassembly kernel. This kernel is closely related to the semantic and spatial information of the input features and can adaptively adjust the upsampling kernel based on the local features at each position. The generated upsampling kernel is normalized using a Softmax operation to ensure that the weights of the upsampling kernel sum to 1, enabling precise weighted processing of the neighboring area for each position. The calculation formula for the reassembly kernel (Equation 1) is as follows:


(1) $$M_{l^{\prime }}=\varphi \left(N(X_{l},K_{encoder})\right)$$

Here, ϕ represents the kernel prediction module, K_encoder_ is the size of the convolution kernel, and N(X_l_, K_encoder_) refers to a local subregion in the feature map centered at position 1, with a size of K_encoder_ × K_encoder​_.

After generating the upsampling kernel, the feature reassembly module applies these kernels to reassemble the input feature map. For each position l′=(x′, y′) in the output feature map, its corresponding position l=(x, y) can be found in the input feature map. The neighborhood N(X_l_, K_up_) around position l is extracted and element-wise multiplied with the upsampling kernel M_l′_, generating the final upsampled feature map X′. The calculation formula for X_l′_ (Equation 2)is as follows:


(2) $$X_{l^{\prime }}=\phi \left(M_{l^{\prime }},N\left(X_{l},K_{up}\right)\right)$$

Here, r represents the radius of the neighborhood, M_l′_(n, m) is the weight at position (n, m) in the upsampling kernel, and X_l_(x+n,y+m) represents the pixel value in the neighborhood of the input feature map.

CARAFE generates the upsampling kernel through content awareness, making the upsampling process no longer reliant on fixed interpolation rules but able to adaptively adjust sampling weights based on the position of the rice leaf tip in the image. This adaptive mechanism ensures precise reconstruction of spatial details and effectively preserves the surrounding semantic information and morphological features of the rice leaf tip. Additionally, the feature of CARAFE reassembly mechanism enhances the semantic representation capability of the feature map through weighted combinations of local regions, ensuring that the positional information and details of the rice leaf tip are fully preserved during the upsampling process, thereby improving the model’s detection performance for very small targets.

#### 2.3.4 Rice leaf tip detection model

**Figure 5:**
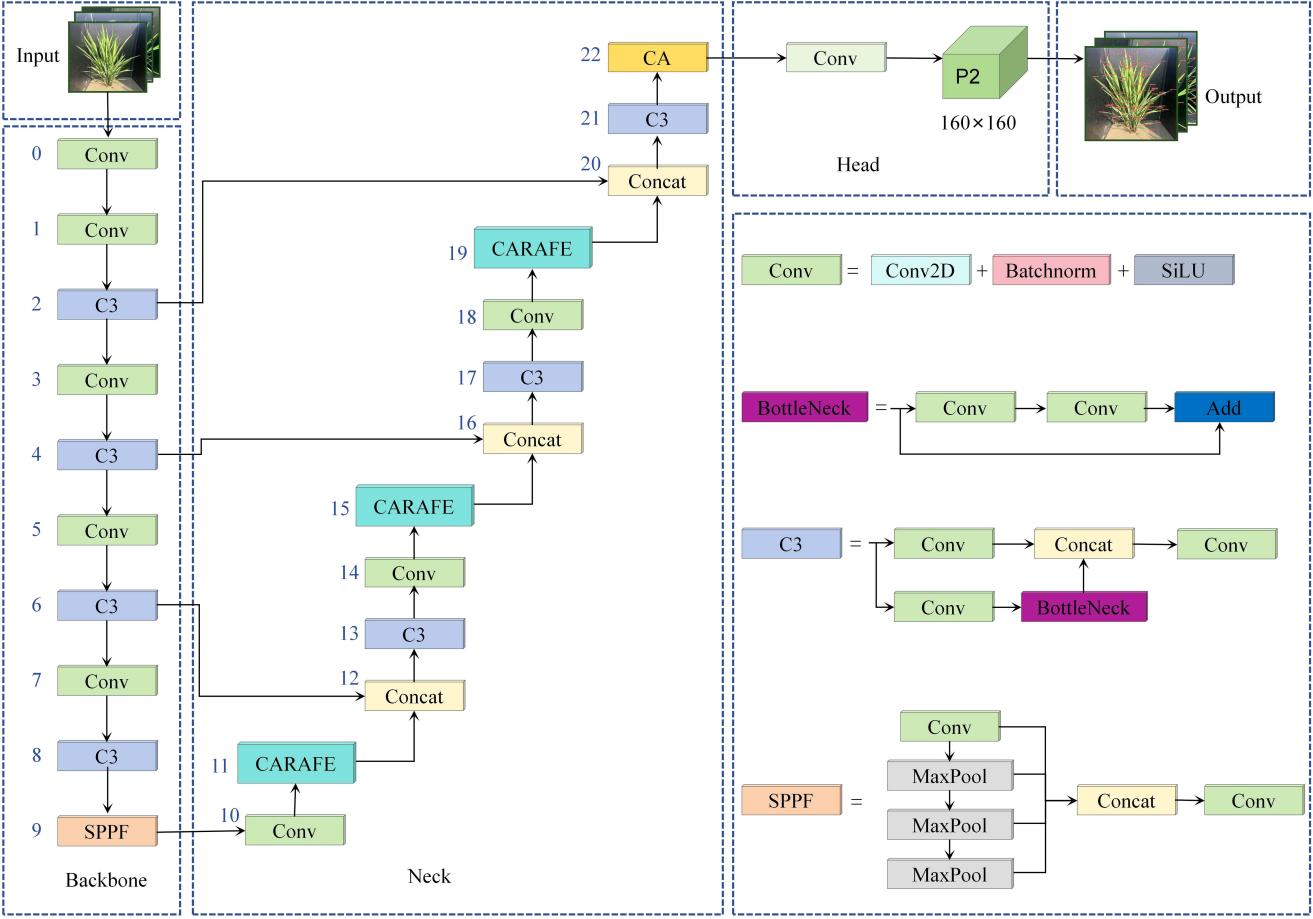
Network structure of rice leaf tip detection model.

**Figure 5** shows the structure of the improved rice leaf tip detection model. The optimized detection layer network introduces a new P2 high-resolution detection layer, effectively capturing the details of small leaf tip targets. The original P3, P4, and P5 low-resolution detection layers are removed, which reduces the loss of small target information and reduces the number of model parameters. To improve the feature extraction and spatial localization capabilities of the P2 high-resolution detection layer further, the CA coordinate attention mechanism is introduced. This mechanism encodes the high-resolution feature map bidirectionally to generate spatial information, effectively improving the model’s ability to represent and localize the features of rice leaf tips, preventing the loss of leaf tip features due to occlusion by other leaves. Finally, by introducing the CARAFE upsampling operator, the sampling weights are adaptively adjusted based on the local information of the feature map, allowing the CA attention mechanism to retain more spatial details and semantic information of the rice leaf tips.

## 3 Results and analysis

### 3.1 Experimental setup and evaluation metrics

#### 3.1.1 Experimental environment and parameters

The experiments in this study were conducted on an Ubuntu 20.04 operating system, with a CPU model of Intel(R) Xeon(R) Platinum 8362 CPU @ 2.80GHz and a GPU model of NVIDIA GeForce RTX 3090 (24GB). The deep learning framework is built using Python 3.8, CUDA 11.3, and PyTorch 1.11.0. The configuration details for training the network model are summarized in **Table 1**.

**Table 1:** Parameter setting.

Parameter	Value
Momentum	0.937
Weight_decay	0.0005
Learning_rate	0.01
Image_size	640
Batch_size	32
Epochs	300
Optimizer	SGD

#### 3.1.2 Evaluation metrics

This study employs Precision (P), Recall (R), Mean Average Precision (mAP), and the number of Parameters as evaluation metrics for the rice leaf tip detection model. The formulas for calculating P (Equation 3), R (Equation 4), and mAP (Equation 5) are as follows.


(3) $$P=\frac{TP}{TP+FP}$$

(4) $$R=\frac{TP}{TP+FN}$$

(5) $$mAP=\frac{1}{N}\sum_{i=1}^{N}\int_{0}^{1}P(R)dR$$

Here, TP represents the count of samples that are correctly predicted as positive by the model. FP refers to the count of samples that are wrongly predicted as positive. FN represents the count of positive samples that the model fails to identify. N denotes the total number of predicted categories by the model, which in this study is 1.

### 3.2 Experimental results and analysis

#### 3.2.1 Comparative analysis of different detection layer design

To analyze the performance of different resolution detection layers in rice leaf tip detection, a comparative experiment was conducted on different detection layer designs. The detection layers, based on the resolution of the corresponding feature maps, are categorized from high to low as P2, P3, P4, and P5. Starting from the combination of P3, P4, and P5 detection layers in the YOLOv5s baseline model, the following operations were tested: adding the P2 detection layer, removing the P5 detection layer, removing the P4 detection layer, and removing the P3 detection layer. The experimental comparison results are shown in **Table 2**.

**Table 2:** Comparison of different detection layer designs.

Detection layer design	Precision P/%	Recall R/%	Mean average Precision mAP/%	Parameters/10^6^
P3+P4+P5	87.2	64.9	75	7.01
P2+P3+P4+P5	89.3	74.4	84.2	7.16
P2+P3+P4	89	74	83.8	5.38
P2+P3	92.3	83	91	4.93
P2	92.5	84.4	92.2	4.81

**Figure 6:**
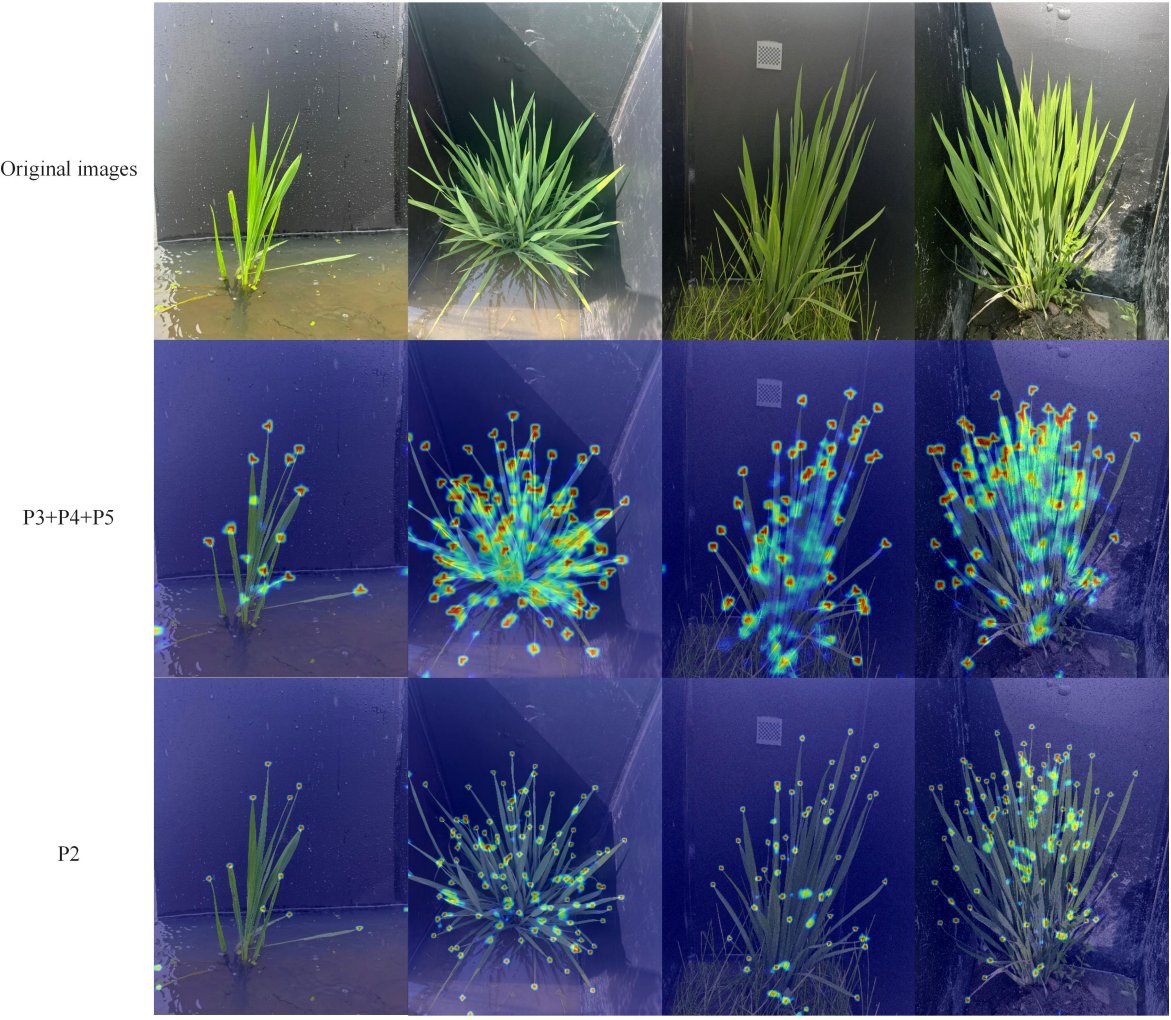
Heatmap.

As shown in **Table 2**, the number of detection layers has a significant impact on the model’s parameter count—the fewer detection layers, the lower the number of parameters. The single P2 high-resolution detection layer design used in this study demonstrates superior performance in all aspects. Compared to the other four detection layer designs, the model with the single P2 high-resolution detection layer improves precision by 5.3%, 3.2%, 3.5% and 0.2%, recall by 19.4%, 10%, 10.4% and 1.4%, and mAP by 17.2%, 8%, 8.4% and 1.2%, respectively. Moreover, while maintaining outstanding detection performance, the model’s parameter count is also significantly reduced by 31.4%, 32.7%, 10.4% and 2.3% compared to the other four designs. To further visualize the enhancement in detection performance with the single high-resolution detection layer design, Grad-CAM (Selvaraju et al., 2019) was used for heatmap analysis, as shown in **Figure 6**. The figure includes four images taken from different periods, angles, and lighting conditions. In the heatmap, the redder the region, the greater its contribution to detection. From the figure, it is evident that the model with the single high-resolution detection layer design has significantly smaller and more focused activation areas, concentrating on the leaf tip regions. This demonstrates a higher dependency on the key features of the leaf tips, showing stronger feature attention capability compared to the baseline model. The model is more optimized in feature selection, effectively suppressing the influence of background and leaf occlusion.

#### 3.2.2 Ablation experiment performance analysis

To further verify the improvements in detection performance brought by the three modifications—single high-resolution detection layer design, CA attention mechanism, and CARAFE upsampling operator—as well as their effectiveness on the rice leaf tip dataset, four sets of ablation experiments were designed. The results are shown in **Table 3**. In these experiments, Model 1 represents the YOLOv5s baseline model. Improvement one refers to the single high-resolution detection layer design, improvement two refers to the addition of the CA attention mechanism, and improvement three refers to the introduction of the CARAFE upsampling operator. In the table, “√” indicates the corresponding modification was applied, while “-” indicates it was not applied.

**Table 3:** Comparison of ablation experiments.

Model	Improvement one	Improvement two	Improvement three	P/%	R/%	mAP/%	Parameters/10^6^
1	–	–	–	87.2	64.9	75	7.01
2	✓	–	–	92.5	84.4	92.2	4.81
3	✓	✓	–	92.4	84.8	92.5	4.82
4	✓	✓	✓	93.7	87	93.5	5.02

As shown in **Table 3**, the single high-resolution detection layer design brings the most significant performance improvement to the model, increasing precision (P), recall (R), and mAP by 5.3%, 19.5% and 17.2%, respectively, while reducing the parameter count by 31.4%. This indicates that rice leaf tips, being extremely small targets, are highly dependent on high-resolution feature maps. After adding the CA attention mechanism, precision slightly decreased, but recall and mAP increased by 0.4% and 0.3%, respectively. This suggests that introducing the CA attention mechanism before the prediction head can enhance the model’s focus on the spatial positioning information of the leaf tips, improving the model’s robustness. When the CARAFE upsampling operator was further introduced, compared to using only the first two modifications, the parameter count slightly increased, but precision, recall, and mAP were further improved by 1.3%, 2.2% and 1%, reaching 93.7%, 87% and 93.5%, respectively. Compared to the YOLOv5s baseline model, these represent increases of 6.5%, 22.1% and 18.5%, respectively, while the parameter count was reduced by 28.4%. This indicates that CARAFE’s adaptive upsampling convolution kernel can effectively retain spatial details and semantic features of rice leaf tips for the high-resolution detection layer and CA attention mechanism. Overall, across the four ablation experiments, using all three improvements together significantly enhances the detection performance of rice leaf tips, notably reducing the miss rate while also reducing the parameter count to some extent.

#### 3.2.3 Performance analysis of different detection models

To further validate the superior performance of the improved rice leaf tip detection model in this study, comparative experiments were conducted between the improved model and the leading object detection models currently in use, including Faster-RCNN, YOLOv7-tiny, and YOLOv8s, under the same environment and using the same dataset. The results are shown in **Table 4**.

**Table 4:** Comparison of different detection models.

Model	P/%	R/%	mAP/%	Parameters/10^6^
Faster R-CNN	42.8	31.1	35.3	41.5
YOLOv5s	87.2	64.9	75	7.01
YOLOv7-tiny	83.1	58.8	67	6.01
YOLOv8s	81.9	60.8	71.1	11.1
Improved YOLOv5s	93.7	87	93.5	5.02

As depicted in **Table 4**, Faster R-CNN achieved a precision, recall, and mAP of only 42.8%, 31.1% and 35.3%, respectively, with a parameter count reaching 41.5M. In terms of both detection performance and parameter count, it is significantly inferior to the one-stage YOLO series algorithms. The improved model in this study achieved a precision of 93.7%, a recall of 87%, and an mAP of 93.5%. Compared to YOLOv5s, YOLOv7-tiny, and YOLOv8s, the improved model increased precision by 6.5%, 10.6% and 11.8%, recall by 22.1%, 28.2%, and 26.2%, and mAP by 18.5%, 26.5% and 22.4%. In terms of parameter count, the model reduced it by 28.4%, 16.5% and 54.8%. The comparison results demonstrate that the improved rice leaf tip detection model in this study delivers optimal detection performance with a relatively low parameter count.

#### 3.2.4 Analysis of model detection performance

**Figure 7:**
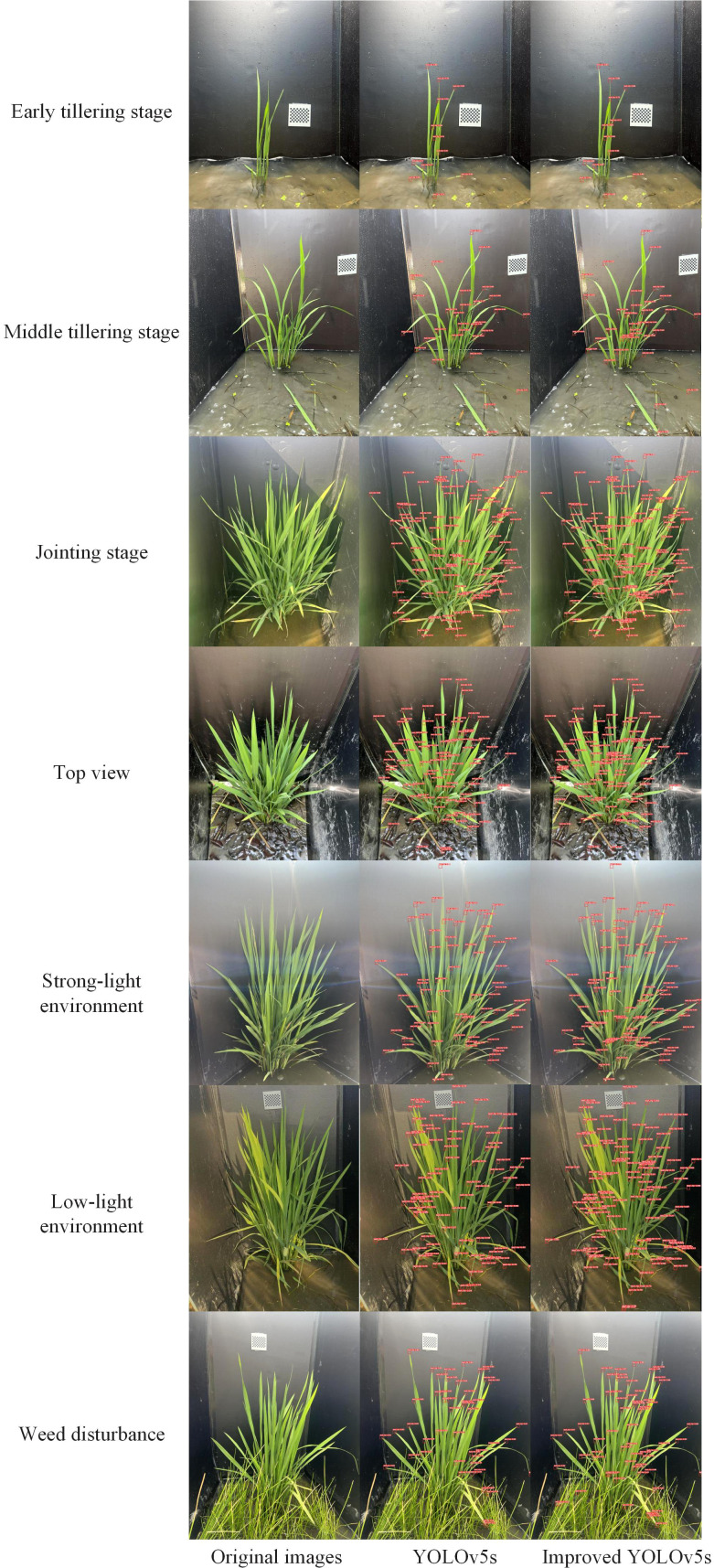
Model detection performance.

To visually demonstrate the effectiveness of the rice leaf tip detection model, we performed leaf tip detection on rice plants under different conditions, including various growth stages, angles, lighting, and interference from weeds. The detection results of the model in this study were compared with those of the YOLOv5s baseline model, as shown in **Figure 7**.

The results indicate that during the early tillering stage, when there are fewer leaves and minimal occlusion, both the baseline model and the model proposed in this study are able to accurately detect all leaf tips. However, as the number of leaves increases in the later tillering stages, leaf overlap and occlusion between plants become more pronounced, leading to a significant increase in missed detections by the baseline model, particularly in the lower parts of the leaves and near the stem, where the leaf tips partially obscured by upper leaves are often undetected. Additionally, under both strong and weak lighting conditions, the baseline model exhibits even more pronounced missed detections. In contrast, the model proposed in this study maintains high detection performance under complex lighting conditions, accurately identifying most leaf tips. Furthermore, in the presence of background interference from weeds, the baseline model fails to detect the occluded leaf tips, whereas the model proposed in this study can still accurately identify leaf tips in obstructed areas, demonstrating stronger background adaptability and robustness.

## 4 Discussion

This study introduces a rice leaf tip detection method based on improved YOLOv5s, which improves the detection accuracy by 18.5% compared with the baseline model while reducing the amount of parameters by 28.4%. This is mainly attributed to the single P2 high-resolution detection layer design proposed in this paper. The reason lies in the fact that the high-resolution detection layer corresponds to high-resolution feature maps, which retain deeper high-semantic information and can provide richer detail for rice leaf tips. Low-resolution feature maps tend to lose local details such as the boundaries and shapes of small targets, leading to inaccurate localization and a reduction in overall recall. By removing the low-resolution detection layers, the model can focus on the key features of the leaf tips in the high-resolution feature maps, reducing redundant calculations. This combination effectively improves the detection accuracy of rice leaf tips while reducing the model’s parameter count. The high-resolution detection layer design is also known as the small target detection layer, Hou et al. (2024) added a small target detection layer to the leaf detection in the seedling stage of wheat, which is similar to that of rice leaves, and fused the coordinate attention mechanism to achieve a final mAP of 85.1%, which significantly improves the detection effect in complex field environments, but with a slight increase in the number of parameters, and only focusing on the seedling stage.

Specifically, one of the strengths of this study is that it was optimized for the needs of leaf tip detection during the whole life cycle, unlike many studies that focus only on the seedling stage or a specific stage of rice. Full-life leaf tip detection can cover the entire growth stage from seedling to maturity, which solves the problem of other methods that have large fluctuations in accuracy at different stages of the rice reproductive cycle.

In addition, many related experiments have adopted potted rice for leaf tip detection, but potted rice is limited by space and has controllable growth conditions, so it usually shows better accuracy in detection. However, field rice is more affected by environmental and climatic factors, and the growth of rice plants is more intricate. Traditional methods often face the problems of poor recognition accuracy and high false detection rate in this environment. Vishal et al. (2020) used YOLOv3 to detect the leaf tips of potted rice and realized automatic counting of rice leaves with a final detection accuracy of 82%. In comparison, the method proposed in this study demonstrated higher detection accuracy in the field environment than in potted rice. Therefore, the present method has a stronger potential for practical application in field crop health monitoring and growth assessment, and can be better adapted to the challenges in natural growing environments. Future research will incorporate environment-aware technology to enhance the stability and adaptability of the model in dynamic and complex environments through multi-source data fusion. In order to solve the problem of leaf occlusion behind rice, future research considers combining the information from multi-view images to enhance the robustness and detection accuracy of the model in occluded environments through the complementarity of different viewpoints.

## 5 Conclusion

This study proposes a new method for detecting rice leaf tips in the field, based on an improved YOLOv5s model, with the aim of enhancing the accuracy and efficiency of rice leaf detection. By introducing a single high-resolution detection layer design, the model’s ability to recognize small targets such as rice leaf tips is strengthened. The incorporation of the CA attention mechanism enhances the model’s focus on rice leaf tip features, even under conditions of weed interference and leaf overlap. Additionally, the use of the CARAFE upsampling operator effectively preserves the spatial details and semantic information of the leaf tips.

1. The improved model achieved precision, recall, and mean average precision (mAP) of 93.7%, 87%, and 93.5%, respectively, representing increases of 6.5%, 22.1% and 18.5% compared to the baseline YOLOv5s model, while also reducing the parameter count by 28.4%. Compared to the current mainstream object detection models, the proposed model demonstrates significant advantages in detection performance. Whether in the early tillering stage with minimal occlusion, or the late tillering and jointing stages with severe leaf overlap, or under varying lighting conditions, different angles, and weed interference, the model shows strong robustness and significantly reduces the missed detection rate of rice leaf tips. This model is capable of quickly and accurately identifying rice leaf tips, providing robust technical support for intelligent monitoring of rice growth, particularly in areas such as leaf counting, health assessment, and fertilization management.
2. In future research, we plan to further optimize the model’s computational resource consumption while maintaining high accuracy, reducing computational costs on low-end devices, and improving its application efficiency on edge devices. Additionally, we aim to integrate data from other sensors to develop a more comprehensive intelligent monitoring system for rice growth, providing all-around technical support for precision agriculture and promoting the application of agricultural automation.

## Data Availability

The raw data supporting the conclusions of this article will be made available by the authors, without undue reservation.
